# Transition of Young People From Beechcroft Child and Adolescent Mental Health Unit to Adult Mental Health Services (AMHS)

**DOI:** 10.1192/bjo.2026.11558

**Published:** 2026-06-30

**Authors:** Katherine Dickson, Claire Kelly

**Affiliations:** Beechcroft Child and Adolescent Mental Health Unit, Belfast, United Kingdom

## Abstract

**Aims:**

The transition of young people who have turned eighteen from Child and Adolescent Mental Health Services (CAMHS) to Adult Mental Health Services (AMHS) is a challenging and high-risk time for patients and their families. There is currently no regional policy for young people transitioning between inpatient services in Northern Ireland. The TRACK Study outlines best practice recommendations in creating a smooth transition for these young people. This audit aims to identify if Beechcroft, Northern Ireland’s Regional CAMHS inpatient Unit, is adherent to these recommendations when transitioning from inpatients to AMHS.

**Methods:**

The electronic records of all Beechcroft inpatients who have turned eighteen and transitioned to AMHS in the past four years were reviewed. Demographics, including diagnosis, length of Beechcroft admission, and time awaiting transfer post-eighteen were collected, as well as data to audit against the four key TRACK recommendations: evidence of transition planning meetings, information transfer, joint working, and continuity of care post-transfer. This allowed us to determine if an optimal transition had taken place for each young person.

**Results:**

Eleven patients were referred from Beechcroft to AMHS and 100% were accepted for a further period of acute care. The majority (91%) were accepted for inpatient care, whilst one young person was accepted by a home-treatment team. Primary diagnoses for young people included psychosis (45%) and depression (36%), with 72% having psychiatric comorbidity. There was a delay in transition of greater than two weeks for 36% of patients. In 100% of cases, adequate information transfer was carried out between CAMHS and AMHS. AMHS attended 91% of transition planning meetings, however, only met with 54% of young people in person prior to transfer. Continuity of care post-transfer, including adult inpatient admission, home treatment, and community mental health team, was maintained for greater than ninety days for 82% of young people.

**Conclusion:**

Young people who turned eighteen in Beechcroft had complex needs, and all required ongoing input from adult acute care. When transitioning patients from Beechcroft to AMHS across Northern Ireland, transition planning meetings and adequate information transfer were consistently carried out. However, we have identified areas for improvement related to transition planning, continuity of care and contact between AMHS and the young person prior to transfer. Results of this audit will be used to develop regional transition guidelines with AMHS to standardise and optimise the transition of young people between CAMHS and AMHS, ensuring the patient remains central throughout this process.

